# The Role of Social Media in Mitigating the Long‐Term Impact of Social Isolation on Mental and Cognitive Health in Older Adults During the COVID‐19 Pandemic: The HUNT Study

**DOI:** 10.1002/gps.70097

**Published:** 2025-05-07

**Authors:** Tanja Louise Ibsen, Ekaterina Zotcheva, Sverre Bergh, Debby Gerritsen, Gill Livingston, Hilde Lurås, Svenn‐Erik Mamelund, Anne Marie Mork Rokstad, Bjørn Heine Strand, Pernille Thingstad, Richard C. Oude Voshaar, Geir Selbæk

**Affiliations:** ^1^ The Norwegian National Centre for Ageing and Health (Ageing and Health) Vestfold Hospital Trust Tønsberg Norway; ^2^ Research Centre for Age‐related Functional Decline and Disease (AFS) Innlandet Hospital Trust Ottestad Norway; ^3^ Department of Primary and Community Care Research Institute for Medical Innovation Radboudum Alzheimer Center Radboud University Nijmegen Medical Center Nijmegen the Netherlands; ^4^ Division of Psychiatry University College London London UK; ^5^ Camden and Islington NHS Foundation Trust London UK; ^6^ Health Services Research Unit Akershus University Hospital Oslo Norway; ^7^ Institute of Clinical Medicine University of Oslo Oslo Norway; ^8^ Centre for Research on Pandemics & Society (PANSOC) at OsloMet ‐ Oslo Metropolitan University Oslo Norway; ^9^ Faculty of Health Sciences and Social Care Molde University College Molde Norway; ^10^ Department of Geriatric Medicine Oslo University Hospital Oslo Norway; ^11^ Department of Physical Health and Ageing Norwegian Institute of Public Health Oslo Norway; ^12^ Department of Neuromedicine and Movement Science Faculty of Medicine and Health Science Norwegian University of Science and Technology Trondheim Norway; ^13^ Department of Health and Social Services Trondheim Municipality Trondheim Norway; ^14^ University of Groningen Groningen the Netherlands; ^15^ Department of Psychiatry University Medical Center Groningen Groningen the Netherlands; ^16^ Institute of Clinical Medicine Faculty of Medicine University of Oslo Oslo Norway

**Keywords:** cognitive function, COVID‐19, HUNT, information and communication technology, longitudinal cohort study, mental health, social isolation, social media

## Abstract

**Background:**

The COVID‐19 pandemic increased social isolation in older adults, promoting mental and cognitive decline. The impact of social media on these effects remains unclear.

**Aim:**

To investigate the long‐term association of social isolation with mental and cognitive health in older adults and whether social media use mitigated this association.

**Method:**

Data from the Norwegian Trøndelag Health Study before (2017–2019), during (January 2021), and after the pandemic (2021–2023) were analysed (*N* = 4844, 53% women, mean age 80 years). Multi‐adjusted mixed‐effects linear regression estimated differences in changes in mental (CONOR‐MHI) and cognitive (MoCA) health related to self‐reported social isolation and social media use. Beta (*β*) represents differences in change in z‐score of CONOR‐MHI or MoCA.

**Results:**

Social isolation was associated with a steeper decline in mental health than no social isolation (*β* = 0.07, 95% CI 0.01, 0.13) but was not associated with change in cognitive health. Daily social media use was not related to change in mental health, whereas it was associated with a less steep cognitive decline than no social media use (< 1 h: *β* = 0.13, 95% CI 0.05, 0.20; ≥ 1 h: *β* = 0.10, 95% CI 0.01, 0.15). Stratified by social isolation, daily social media use < 1 h was related to a less steep cognitive decline than no social media use in both isolated (*β* = 0.15, 95% CI 0.02, 0.28) and non‐isolated individuals (*β* = 0.13, 95% CI 0.03, 0.22).

**Conclusion:**

Individuals experiencing social isolation during the pandemic had a steeper decline in mental, but not cognitive health, compared to those not isolated. Social media use did not buffer the decline in mental health but was associated with less steep cognitive decline. The pandemic showed limits of relying solely on digital solutions for social contact.

**Trial Registration:**

The study is registered in ClinicalTrials.gov 18.02.2021, with the identification number NCT 04792086

**Summary**

- Those experiencing social isolation during the pandemic faced a larger decline in mental health, but not cognitive health, compared to those who were not isolated.
- Staying connected through social media during the pandemic did not prevent mental health decline but was associated with a slower rate of cognitive decline in both socially isolated and not isolated individuals.
- The COVID‐19 pandemic highlighted the limitations of relying only on digital solutions to maintain social connections, mental health, and cognitive function.

## 1 Background

The COVID‐19 pandemic profoundly affected public health, particularly due to the high levels of social isolation experienced by many during the pandemic. Widespread lockdowns, physical distancing measures, and restrictions on social gatherings were considered essential for limiting the spread of SARS‐CoV‐2. Numerous studies have documented that prolonged social isolation was associated with severe consequences for mental health, physical function, and cognitive abilities, especially in vulnerable groups such as older adults and individuals with pre‐existing health conditions [1, 2, 3]. There was a notable increase in mental health challenges such as depression, stress‐related disorders, and loneliness during the pandemic [4, 5, 6, 7, 8]. Furthermore, prior research links social isolation with cognitive decline, as engagement in social activities appears to play a protective role against cognitive deterioration in older adults [9, 10]. However, loneliness and cognitive decline may interact bidirectionally, potentially influencing each other over time [11]. A recent review found that loneliness and social isolation during COVID‐19 correlated with cognitive decline among home‐dwelling older adults and those admitted to nursing homes [12].

Communication through social media during the COVID‐19 crisis may have served as a buffer against the adverse effects of isolation. Virtual communication platforms allow people to maintain social connections despite physical restrictions, raising questions about the extent to which these digital interactions could alleviate feelings of isolation and preserve mental and cognitive health. Some research suggests that social media contributed to reducing social isolation and psychological distress in older adults during the pandemic, as it made it possible to maintain contact with friends, do internet shopping, and keep in contact with healthcare services [13, 14, 15, 16]. In contrast, a recent study found that time spent on social media was associated with higher levels of mental distress among individuals who used social media to reduce loneliness [17]. Social media has also been associated with positive mental health outcomes among those in their sixties but not among those 70 years or older [18].

In addition to the direct effects of social media on both social isolation and mental health, social media use may also moderate the association between social isolation and mental health. A study on relationships and depression during the COVID‐19 lockdown found that daily face‐to‐face or phone/video contact was associated with lower depressive symptoms compared to having no contact [19]. A Chinese study found that less frequent internet use strengthened the association between social isolation and poorer cognitive functioning and higher levels of depression, compared to more frequent internet usage [20]. However, the rate of internet use among older adults in China is low (11.4%) compared to that in Western countries, such as Norway (84%) [21]. This highlights the need for further research on the role of social media on mental and cognitive health in Western regions.

The COVID‐19 pandemic provides a unique context to explore how enforced social isolation affects mental health and cognitive abilities, as well as to evaluate the role of social media in mitigating these effects. Understanding these relationships is critical for informing future public health strategies, particularly in preparing for similar crises that require social distancing measures. In this study we aimed to investigate whether social isolation among older adults during the COVID‐19 pandemic was associated with long‐term mental and cognitive health outcomes. Additionally, we explored how daily use of social media related to changes in mental and cognitive health in both individuals who experienced social isolation and those who did not, utilizing longitudinal data collected before, during, and after the pandemic.

## 2 Material and Methods

### 2.1 Study Design and Participants

**FIGURE 1:**
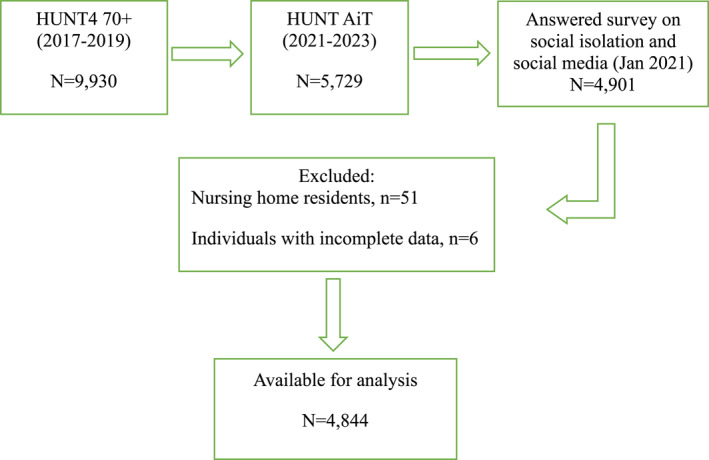
Flow chart participants inclusion.

We used a longitudinal population‐based cohort of participants aged ≥ 70 years from the Norwegian Trøndelag Health Study (HUNT4 70+, 2017–2019), a questionnaire on social isolation and use of social media administrated in the same population during the pandemic (January 2021), and a follow‐up study, HUNT Ageing in Trøndelag (HUNT AiT, 2021–2023). HUNT is a population‐based study that has invited the entire adult population of North Trøndelag to participate in four waves, the first time in 1984 [22]. The participants answered questionnaires that included socio‐demographic and clinical data, and attended a comprehensive clinical evaluation by health professionals at field stations (84%), participants' own homes (8%), or nursing homes (8%) [23]. HUNT4 70+ included 9930 individuals from North Trøndelag, of which 5729 participated in HUNT AiT. Of these, 4901 had completed the questionnaire on social isolation and social media in January 2021. For the present study, we excluded nursing home residents (*n* = 51) and individuals with incomplete data (*n* = 6). Excluded participants were older (mean age: 82.5 vs. 80 years). A total of 4844 participants provided data for the study (see flow chart Figure 1).

## 3 Data Collection

We included data from HUNT4 70+ and HUNT AiT on sex, year of birth, education (primary, secondary, and tertiary), cohabitation status (alone or with others), and cognitive and mental health statuses (variables described below). Sociodemographic factors and mental health status were self‐reported, while trained health professionals assessed participants' cognitive status [22, 23]. The survey on social isolation and social media (variables described below) was conducted through a postal questionnaire and returned using a provided pre‐paid envelope [24].

### 3.1 Outcome Measures

Mental health was assessed using the CONOR Mental Health Index (CONOR‐MHI), which includes seven items rated on a 1–4‐point scale (total score range: 7–28). The items evaluate feelings such as being nervous and unsettled, troubled by anxiety, secure and calm, irritable, happy and optimistic, sad/depressed, and lonely. An average score was calculated across all seven items, resulting in a range of 1–4, with a threshold of ≥ 2.15 indicating potential mental health challenges [25]. For missing data, individual missing values were replaced with the sample mean for that specific item, whereas records with two or more missing responses were excluded from the analysis [25]. Higher scores indicate worse mental health.

Cognitive function was assessed using the Montreal Cognitive Assessment (MoCA), a comprehensive 30‐point tool designed to evaluate cognitive status across six key domains: visuospatial construction, executive function, episodic memory, attention, language, and orientation [26]. Originally, a cutoff score of 26/30 was suggested. However, a recent meta‐analysis found that a cutoff score of 23/30 provides the best diagnostic accuracy for cognitive impairment [27].

### 3.2 Exposures and Covariates

Social isolation was defined as a ‘lack of social contact with others’ and was assessed based on participants' self‐report on experienced social isolation (yes/no) with the question; During the coronavirus pandemic, did you experience social isolation? [24].

To assess the use of communication through social media participants were asked to report the amount of time they spent daily using screen‐based media to connect with friends or engage with social networks during the pandemic; no use, < 1 h, 1–3 h, or more than 3 h [21]. As there were few participants (*n* = 65) in our study reporting using social media more than 3 h daily, we collapsed the categories ‘1–3 h’ and ‘more than 3 h’ to one category named ‘1 h or more’.

## 4 Statistical Analysis

Data analysis was conducted using STATA 18 software [28]. Sample characteristics are reported as means with standard deviations (SD) for continuous variables and as frequencies with percentages for categorical variables. The main analyses were conducted separately for the two outcomes: mental health and cognitive function. Changes in assessment scores for mental health and cognitive function between baseline and follow‐up were analysed using a multilevel mixed‐effects linear regression model with random intercept and random slope. Mental health and cognitive function served as outcome variables whereas social isolation and social media use were treated as the exposure variables in separate models. A dichotomous time variable was included (baseline at HUNT4 70+/follow‐up at AiT), and an interaction term between time and social isolation was incorporated to evaluate the long‐term association of social isolation with changes in mental health and cognitive function from baseline to follow‐up. Similarly, an interaction term between time and social media use was included in a separate model to evaluate the long‐term association of social media use with change in mental health and cognitive function. To assess the association of social media use with change in mental and cognitive health separately in individuals who experienced social isolation and those who did not, analyses for both outcome variables were repeated stratified by social isolation (isolated vs. not isolated), with social media use as the exposure variable. All models were adjusted for the potential confounders sex, age, education level, and cohabitation status. For comparative purposes, both outcome measures were standardized by subtracting their respective mean values and divided by their standard deviation for each measurement, providing *z*‐scores, with beta (*β*) representing the amount of change in the *z*‐score for the outcome measure. Both time points were included in the standardization. Statistics for the multilevel mixed‐effects linear regression models, with standardized regression coefficients, are reported in Supporting Information S1: Tables 1 and 2.

## 5 Results

The study sample consisted of 4844 participants, with a mean age of 80 years (SD: 5.65, range: 74–100 years). Women represented 53% of the sample. Participant characteristics are presented in Table 1.

**TABLE 1:** Description of the study sample that completed follow‐up (HUNT‐AiT), including baseline characteristics,^a^ stratified by isolation status during the pandemic.

Study sample	Total *N* = 4844 *n* (%)	Isolated *n* = 1831 (38.0%) *n* (%)	Not isolated *n* = 2986 (62.0%) *n* (%)
Sex^b^			
Women	2579 (53.2)	1147 (62.6)	1415 (47.4)
Men	2265 (46.8)	684 (37.4)	1571 (52.6)
Age, mean (SD)	80 (4.6)	80 (4.7)	80 (4.6)
Age baseline	78 (4.6)	76 (4.7)	76 (4.5)
Education^b^ (*n* = 4841)			
Primary	1109 (22.9)	435 (23.8)	669 (22.4)
Secondary	1175 (24.3)	453 (24.8)	718 (24.1)
Tertiary	2557 (52.8)	941 (51.5)	1598 (53.5)
Cohabitation status (*n* = 4807)			
Living alone	1573 (33.6)	613 (33.8)	701 (23.6)
Living with someone	3111 (66.4)	1201 (66.2)	2266 (76.4)
Cohabitation status baseline			
Living alone	1321 (27.5)	721(40.9)	841 (29.1)
Living with someone	3486 (72.5)	1043 (59.1)	2052 (70.9)
Social media^c^ (*n* = 4750)			
No time	639 (13.5)	215 (12.0)	430 (14.3)
Less than 1 hour	2771 (58.3)	1036 (57.7)	1719 (58.7)
1 hour or more	1340 (28.2)	544 (30.3)	789 (27.0)
Mental health^d^ (*n* = 4299), mean (SD)	1.47 (0.40)	1.59 (0.43)	1.40 (0.36)
Mental health baseline	1.41 (0.38)	1.50 (0.42)	1.35 (0.34)
MoCA^e^ score (*n* = 4777), mean (SD)	22.96 (4.12)	22.72 (4.32)	23.12 (3.99)
MoCA baseline	24.07 (3.47)	23.91 (3.71)	24.17 (3.31)

^a^ Baseline data on those participating in the follow‐up, HUNT AiT.

^b^ Sex and education was only collected at baseline, HUNT4 70+.

^c^ Sosial media use was collected during the pandemic, January 2021.

^d^ Mental health (CONOR‐MHI), range 1–4.

^e^ MoCA= Montreal Cognitive Assessment score ranges from 0 to 30 points.

### 5.1 Mental Health

**FIGURE 2:**
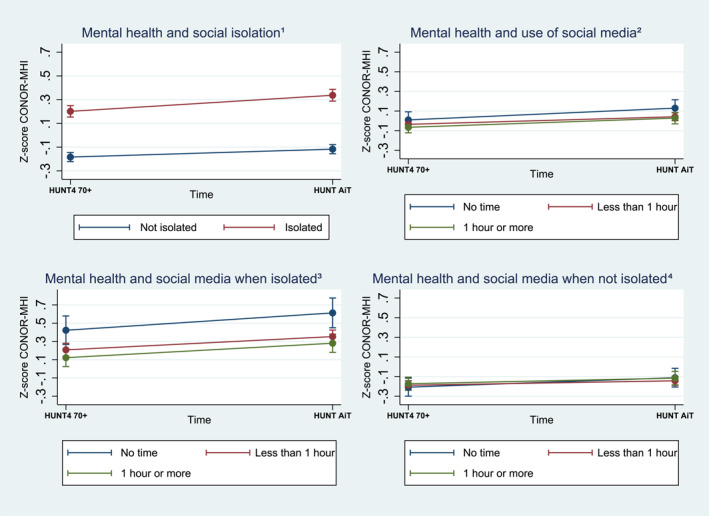
The association between mental health decline (higher score on CONOR‐MHI) and ^1^social isolation, the ^2^use of social media, and the ^3,4^use of social media by isolation status during the pandemic from baseline to follow‐up, with 95% confidence intervals (95% CI), calculated using multilevel mixed‐effects linear regression with standardized regression coefficient, adjusted for sex, age, education level, and cohabitation status.

There was an increase in standardized mental health scores (CONOR‐MHI) in the whole study sample from baseline to follow up after the pandemic (*β* = 0.09, 95% CI 0.05, 0.12) indicating a decline in mental health (Supporting Information S1: Table 1). Those who experienced social isolation during the pandemic had a more severe decline in standardized mental health scores after the pandemic than those who did not experience isolation (*β* = 0.07, 95% CI 0.01, 0.13). The use of social media during the pandemic was not associated with changes in mental health scores from baseline to follow‐up, regardless of whether individuals experienced isolation during the pandemic or not (Figure 2, Supporting Information S1: Table 1).

### 5.2 Cognitive Function

**FIGURE 3:**
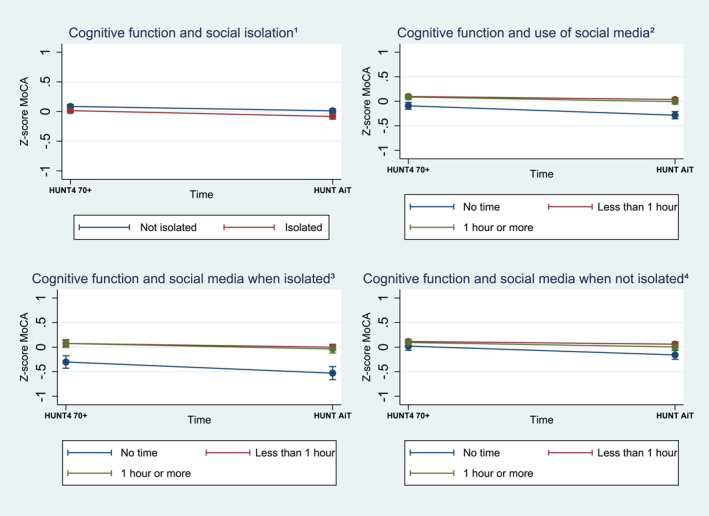
The association between decline in cognitive function (lower score on MoCA) and ^1^social isolation, the ^2^use of social media, and the ^3,4^use of social media by isolation status during the pandemic from baseline to follow‐up, with 95% confidence intervals (95% CI), calculated using multilevel mixed‐effects linear regression with standardized regression coefficient, adjusted for sex, age, education level, and cohabitation status.

There was a decrease in standardized cognitive function scores (MoCA) in the whole study sample from baseline to follow up after the pandemic (*β* = −0.08, 95% CI −0.11, −0.05) (Supporting Information S1: Table 2). Social isolation was not associated with changes in cognitive function from baseline to follow‐up. Daily use of social media less than 1 h during the pandemic (*β* = 0.13, 95% CI 0.05, 0.20) or 1 h or more (*β* = 0.10, 95% CI 0.01, 0.18) was associated with less steep cognitive decline, compared to those who did not use social media (Figure 3, Supporting Information S1: Table 2).

Analysis stratified by isolation status showed that using social media less than 1 h a day was associated with a less steep cognitive decline compared to no daily social media use, for both those who experienced social isolation (*β* = 0.15, 95% CI 0.02, 0.28) and those who did not (*β* = 0.13, 95% CI 0.03, 0.22). There was a similar trend among those who used social media for 1 h or more, but this difference was not statistically significant (Figure 3, Supporting Information S1: Table 2).

## 6 Discussion

The present study found that there was a decline in mental health and cognitive function among older Norwegian adults during the COVID‐19 pandemic. Those who experienced social isolation during the pandemic had a more severe decline in mental health scores during the pandemic than those who were not isolated. The decline in mental health was not mitigated by using social media for contact with others, for either the isolated or the non‐isolated group. We found no association between social isolation and the decline in cognitive function. However, there was an association between using social media, irrespective of time use, and a less steep decline in cognitive function in the entire study sample. However, the association between social media use and less steep cognitive decline was most pronounced for those using social media less than 1 h daily, compared to those not using social media at all. This association remained consistent regardless of whether the participants experienced social isolation during the pandemic or not.

### 6.1 Mental Health

The decline in mental health and the link between social isolation and reduced mental health during the pandemic was expected, as it is well documented in earlier research [4, 7]. Over the past decade, society has sought effective measures to prevent social isolation leading to mental distress. Among these efforts, considerable attention has been devoted to developing digital strategies that empower older adults to build and maintain social relationships using social media [29, 30]. One of the barriers for success is the lack of digital competence among older adults [29, 31, 32]. In our study, 14% of the participants did not use social media, suggesting a need for additional digital training if social media should help them build and maintain relationships. On the other hand, as many as 86% of the participants in our study used social media to stay connected with others during the pandemic. Regardless of this, social media use did not mitigate the decline in mental health scores.

Based on our findings, social media alone does not appear to be a sufficient measure to prevent the deterioration of mental health challenges among socially isolated older adults. This may be seen in relation to earlier research pointing out that those who actively used social media to reduce loneliness during the pandemic, showed higher scores on psychological distress [17]. Although social isolation (lack of social contact with, or separation from, family and friends) and loneliness (a subjective feeling of lack of connectedness with others) are distinct concepts, they are associated with each other [12]. Two reviews [15, 16] and a cross‐sectional study [13] reported that using social media had positive effects on mental health during the pandemic. However, the studies referenced in the reviews were conducted during the early phase of the pandemic (2020, and one in 2021) [15, 16]. Similarly, the cross‐sectional study was conducted in June and July 2020 [13]. This timing may account for the differences in findings compared to our longitudinal study, which includes data collection both before and after the pandemic. Another important consideration is that the positive effects of social media use on mental health may depend on how it is utilized, having more positive effects when used for communication among an established network [13], integrated into interventions such as communication with caregivers, or participation in organized online activities [16, 30]. Further, studies conducted during the pandemic indicate that the use of social media had a less positive impact on mental health compared to face‐to‐face interactions [33, 34].

### 6.2 Cognitive Function

The present study documented cognitive decline in older adults during the pandemic, which was anticipated due to the advanced age of the participants and the 4‐year interval between baseline and follow‐up. However, it was unexpected that social isolation did not impact changes in cognitive function, as the link between social isolation and changes in cognitive function is well‐documented in previous research [9, 10, 12]. This is likely explained by the period of isolation, limited to the duration of the pandemic, which may have been too short to exhibit the patterns observed in earlier studies.

Existing research reports that social relations have a positive impact on cognitive function [9, 10]. Social interaction likely serves as cognitive stimulation and contributes to building cognitive reserve, which supports the maintenance of brain function [9]. Thus, contact with others via social media can play a significant role in maintaining social activity and providing cognitive engagement [20]. In our study sample any amount of daily social media use was associated with a less steep cognitive decline. This is in line with earlier research reporting that contact with others, even when it is through social media rather than face‐to‐face interaction, can stimulate cognitive function [20, 35].

Interestingly, the significance of social media use on cognitive function was smaller in the stratified analysis by isolation status. There, daily social media use for 1 h or more had a less prominent, and not statistically significant, impact on mitigating cognitive decline, regardless of whether the participants experienced isolation during the pandemic or not, whereas daily social media use for less than 1 hour was still associated with a less steep cognitive decline in both groups. One possible explanation may be that online interactions may lack the emotional depth and social support needed to effectively stimulate and sustain cognitive health. Thus, extended daily social media use did not offer the same level of benefit on cognitive function as less use. Recent research suggests that the COVID‐19 pandemic has increased the frequency of digital device use across all age groups, contributing to more media multitasking. However, it appears that this has a less positive effect on cognition in older adults compared to younger individuals (< 29 years). Additionally, social media use might replace other cognitively stimulating activities [36]. Consequently, positive associations between cognitive function and social media use may reach a plateau at around 1 h per day, with higher use not providing any further cognitive benefits.

## 7 Strengths and Limitations

A key strength of this study is its utilization of individual longitudinal data from a large, population‐based survey. Since the data on mental health and cognitive function were collected immediately before the pandemic (HUNT4 70+) and then again after its onset (HUNT AiT), the risk of recall bias is minimal. However, the data concerning social isolation and social media use were collected one year after the COVID‐19 outbreak, which may have introduced memory‐related biases. Furthermore, social isolation was measured with only one dichotomous question. There may be differences in how the participants interpreted the question on ‘contacting friends and other networks' using social media. Thus, comparison with other studies on how social media use during the pandemic is associated with mental health and cognitive function must be interpreted with caution. We cannot determine the direction of the associations in our findings; therefore, individuals who experienced cognitive decline or a decline in mental health may have used social media less because it was more challenging for them to use. Similarly, they may have experienced social isolation simply due to an already initiated decline in cognitive or mental health. Furthermore, we did not adjust for lifestyle‐related factors (e.g. smoking, alcohol use, and activity of daily living), as these may act as mediators rather than confounders, and adjusting for them could underestimate the total effect of social isolation. All participants in the study were residents of central Norway, which may not reflect the demographics of other regions in Norway or populations internationally. Also, the high baseline level of internet use in the sample (84%) may limit the generalizability of the findings to populations with lower digital literacy or limited access to technology. Moreover, the sample consisted primarily of individuals of Norwegian ethnicity, restricting the generalizability of the findings to other ethnic groups [22].

## 8 Conclusion

Older Norwegian adults experienced mental and cognitive decline during the COVID‐19 pandemic. Social isolation during the pandemic was linked to a more severe decline in mental health, while no such association was found between social isolation and cognitive function. Social media use did not mitigate the mental health decline but was associated with a less steep cognitive decline. Using social media for less than 1 hour a day yielded the most prominent mitigation in cognitive decline, both in those who experienced social isolation, and those who did not. This suggests that while social media can provide some cognitive benefits, it is not sufficient for addressing mental health challenges posed by social isolation. The increasing digitalization of society may raise expectations for digital strategies to address challenges caused by social isolation. However, the COVID‐19 pandemic has further underscored these challenges, highlighting the limitations of solely depending on digital solutions for maintaining social contact. Our findings highlight the necessity for comprehensive approaches that integrate social media with other forms of social support. Such strategies are essential to build and maintain relationships established in various settings and to enhance both mental health and cognitive function in older adults.

## Author Contributions

G.S. led the study project and, together with B.H.S., S.B., and T.L.I., was responsible for the study's concept and design. E.Z., B.H.S., and T.L.I. carried out the analysis. Interpretation of the data was a collaborative effort involving T.L.I., E.Z., B.H.S., S.B., D.G., G.L., H.L., S.E.M., R.C.O.V., A.M.M.R., P.T., G.S., and T.L.I. draughted the manuscript, with substantial contributions from all authors in revising the work. All authors reviewed and approved the final version of the manuscript.

## Ethics Statement

This study was approved by the Regional Committee for Medical and Health Research Ethics in Norway (REK Southeast B, reference number 182575). All procedures adhered to REK's guidelines and aligned with the principles outlined in the Declaration of Helsinki. It forms part of a broader project registered on ClinicalTrials.gov (ID: NCT 04792086). Informed written consent was obtained from all participants in the HUNT4 70+ study. For those with limited capacity to provide consent, their next of kin granted permission on their behalf. The consent form explicitly stated that collected data could be linked to other registries for use in approved research projects, as was done in this study.

## Conflicts of Interest

The authors declare no conflicts of interest.

## Supporting information

Supporting Information S1

## Data Availability

The data underlying this study's findings are sourced from the HUNT database. However, due to licencing restrictions, they are not publicly available. Access to the data may be granted upon reasonable request to the authors, upon to approval from the HUNT database.

## References

[1] J. Holt‐Lunstad , T. B. Smith , M. Baker , T. Harris , and D. Stephenson , “Loneliness and Social Isolation as Risk Factors for Mortality: A Meta‐Analytic Review,” Perspectives on Psychological Science 10, no. 2 (2015): 227–237, 10.1177/1745691614568352. 25910392

[2] Z. I. Santini , P. E. Jose , E. York Cornwell , et al., “Social Disconnectedness, Perceived Isolation, and Symptoms of Depression and Anxiety Among Older Americans (NSHAP): A Longitudinal Mediation Analysis,” Lancet Public Health 5, no. 1 (2020): e62–e70, 10.1016/s2468-2667(19)30230-0. 31910981

[3] N. K. Valtorta , M. Kanaan , S. Gilbody , and B. Hanratty , “Loneliness, Social Isolation and Risk of Cardiovascular Disease in the English Longitudinal Study of Ageing,” European Journal of Preventive Cardiology 25, no. 13 (2018): 1387–1396, 10.1177/2047487318792696. 30068233

[4] L. Knox , G. C. Karantzas , D. Romano , J. A. Feeney , and J. A. Simpson , “One Year on: What We Have Learned About the Psychological Effects of COVID‐19 Social Restrictions: A Meta‐Analysis,” Current Opinion in Psychology 46 (2022): 101315, 10.1016/j.copsyc.2022.101315. 35398753 PMC8907153

[5] B. Pfefferbaum and C. S. North , “Mental Health and the Covid‐19 Pandemic,” New England Journal of Medicine 383, no. 6 (2020): 510–512, 10.1056/NEJMp2008017. 32283003

[6] J. Ruan , Y. M. Xu , and B. L. Zhong , “Loneliness in Older Chinese Adults Amid the COVID‐19 Pandemic: Prevalence and Associated Factors,” Asia‐Pacific Psychiatry 15, no. 4 (2023): e12543, 10.1111/appy.12543. 37562972

[7] C. Silva , C. Fonseca , R. Ferreira , et al., “Depression in Older Adults During the COVID‐19 Pandemic: A Systematic Review,” Journal of the American Geriatrics Society 71, no. 7 (2023): 2308–2325, 10.1111/jgs.18363. 37029710

[8] Y. Xiong , H. Hong , C. Liu , and Y. Q. Zhang , “Social Isolation and the Brain: Effects and Mechanisms,” Molecular Psychiatry 28, no. 1 (2023): 191–201, 10.1038/s41380-022-01835-w. 36434053 PMC9702717

[9] M. E. Kelly , H. Duff , S. Kelly , et al., “The Impact of Social Activities, Social Networks, Social Support and Social Relationships on the Cognitive Functioning of Healthy Older Adults: A Systematic Review,” Systematic Reviews 6, no. 1 (2017): 259, 10.1186/s13643-017-0632-2. 29258596 PMC5735742

[10] J. S. Kuiper , M. Zuidersma , S. U. Zuidema , et al., “Social Relationships and Cognitive Decline: A Systematic Review and Meta‐Analysis of Longitudinal Cohort Studies,” International Journal of Epidemiology 45, no. 4 (2016): 1169–1206, 10.1093/ije/dyw089. 27272181

[11] B. L. Zhong , S. L. Chen , X. Tu , and Y. Conwell , “Loneliness and Cognitive Function in Older Adults: Findings From the Chinese Longitudinal Healthy Longevity Survey,” Journals of Gerontology Series B: Psychological Sciences and Social Sciences 72, no. 1 (2017): 120–128, 10.1093/geronb/gbw037. 27013536 PMC5156491

[12] K. Kassam and J. M. McMillan , “The Impact of Loneliness and Social Isolation During COVID‐19 on Cognition in Older Adults: A Scoping Review,” Frontiers in Psychiatry 14 (2023): 1287391, 10.3389/fpsyt.2023.1287391. 38045621 PMC10690360

[13] A. Hajek and H. H. König , “Frequency of Contact With Friends and Relatives Via Internet and Psychosocial Factors in Middle‐Aged and Older Adults During the COVID‐19 Pandemic. Findings From the German Ageing Survey,” International Journal of Geriatric Psychiatry 37, no. 1 (2022), 10.1002/gps.5623. PMC8646763 34505322

[14] S. Matovic , S. Grenier , F. Jauvin , et al., “Trajectories of Psychological Distress During the COVID‐19 Pandemic Among Community‐Dwelling Older Adults in Quebec: A Longitudinal Study,” International Journal of Geriatric Psychiatry 38, no. 1 (2023): e5879, 10.1002/gps.5879. 36703303

[15] S. Rivera‐Torres , E. Mpofu , M. Jean Keller , and S. Ingman , “Older Adults' Mental Health Through Leisure Activities During COVID‐19: A Scoping Review,” Gerontol Geriatr Med 7 (2021): 23337214211036776, 10.1177/23337214211036776. 34395816 PMC8361539

[16] N. G. Rodrigues , C. Q. Y. Han , Y. Su , P. Klainin‐Yobas , and X. V. Wu , “Psychological Impacts and Online Interventions of Social Isolation Amongst Older Adults During COVID‐19 Pandemic: A Scoping Review,” Journal of Advanced Nursing 78, no. 3 (2022): 609–644, 10.1111/jan.15063. 34625997 PMC8661520

[17] H. Ragnhildsløkken , T. Bonsaksen , E. Aakhus , et al., “Social Media Use and Associations With Psychological Distress Among Older Adults During the COVID‐19 Pandemic,” Social Sciences 13, no. 12 (2024): 634, 10.3390/socsci13120634.

[18] T. Bonsaksen , H. Thygesen , J. Leung , et al., “Video‐Based Communication and Its Association With Loneliness, Mental Health and Quality of Life Among Older People During the COVID‐19 Outbreak,” International Journal of Environmental Research and Public Health 18, no. 12 (2021): 6284, 10.3390/ijerph18126284. 34200670 PMC8296058

[19] A. Sommerlad , L. Marston , J. Huntley , et al., “Social Relationships and Depression During the COVID‐19 Lockdown: Longitudinal Analysis of the COVID‐19 Social Study,” Psychological Medicine 52, no. 15 (2021): 1–10, 10.1017/s0033291721000039. PMC7844174 33436126

[20] Y. Li , X. Bai , and H. Chen , “Social Isolation, Cognitive Function, and Depression Among Chinese Older Adults: Examining Internet Use as a Predictor and a Moderator,” Frontiers in Public Health 10 (2022): 809713, 10.3389/fpubh.2022.809713. 35359786 PMC8963936

[21] S. Eriksen , A. M. M. Rokstad , G. Selbæk , et al., “Use of Screen‐Based Media Among Older People During the COVID‐19 Pandemic. A HUNT Study,” Sykepleien forskning 17, no. 88131 (2022), 88131, 10.4220/Sykepleienf.2022.88131en.

[22] B. O. Åsvold , A. Langhammer , T. A. Rehn , et al., “Cohort Profile Update: The HUNT Study, Norway,” International Journal of Epidemiology 52, no. 1 (2023): e80–e91, 10.1093/ije/dyac095. 35578897 PMC9908054

[23] L. Gjøra , B. H. Strand , S. Bergh , et al., “Current and Future Prevalence Estimates of Mild Cognitive Impairment, Dementia, and Its Subtypes in a Population‐Based Sample of People 70 Years and Older in Norway: The HUNT Study,” Journal of Alzheimer's Disease 79, no. 3 (2021): 1213–1226, 10.3233/jad-201275. PMC7990439 33427745

[24] T. L. Ibsen , A. M. M. Rokstad , S. Eriksen , et al., “Sosial Isolasjon Blant Eldre Under Koronapandemien [Social Isolation Among Older Adults During the COVID‐19 Pandemic],” Forlaget aldring og helse ‐akademisk (2022), https://butikk.aldringoghelse.no/file/digitalarkiv‐nettbutikk/sosial‐isoalsjon‐blant‐eldre‐under‐koronapandemien.pdf.

[25] Søgaard, A. J. , Bjelland, I. , Tell, G. S. , & Røysamb, E. , “A Comparison of the CONOR Mental Health Index to the HSCL‐10 and HADS: Measuring Mental Health Status in The Oslo Health Study and the Nord‐Trøndelag Health Study,” Norsk Epidemiologi, 13, no. 2 (2003): 279–284, 10.5324/nje.v13i2.296.

[26] Z. Nasreddine , N. Phillips , V. Bédirian , et al., “The Montreal Cognitive Assessment, MoCA: A Brief Screening Tool for Mild Cognitive Impairment,” JAGS 53 (2005): 695–699. 10.1111/j.1532-5415.2005.53221.x 15817019

[27] N. Carson , L. Leach , and K. J. Murphy , “A Re‐Examination of Montreal Cognitive Assessment (MoCA) Cutoff Scores,” International Journal of Geriatric Psychiatry 33, no. 2 (2018): 379–388, 10.1002/gps.4756. 28731508

[28] StataCorp , Stata Statistical Software: Release 18 (StataCorp LLC, 2023).

[29] S. Baker , J. Warburton , J. Waycott , et al., “Combatting Social Isolation and Increasing Social Participation of Older Adults Through the Use of Technology: A Systematic Review of Existing Evidence,” Australasian Journal on Ageing 37, no. 3 (2018): 184–193, 10.1111/ajag.12572. 30022583

[30] H. K. Choi and S. H. Lee , “Trends and Effectiveness of ICT Interventions for the Elderly to Reduce Loneliness: A Systematic Review,” Healthcare 9, no. 3 (2021): 293, 10.3390/healthcare9030293. 33800099 PMC8002106

[31] N. A. Ahmad , M. F. Abd Rauf , N. N. Mohd Zaid , A. Zainal , T. S. Tengku Shahdan , and F. H. Abdul Razak , “Effectiveness of Instructional Strategies Designed for Older Adults in Learning Digital Technologies: A Systematic Literature Review,” SN Computer Science 3, no. 2 (2022): 130, 10.1007/s42979-022-01016-0. 35039803 PMC8754191

[32] V. Welch , E. T. Ghogomu , V. I. Barbeau , et al., “Digital Interventions to Reduce Social Isolation and Loneliness in Older Adults: An Evidence and Gap Map,” Campbell Systematic Reviews 19, no. 4 (2023): e1369, 10.1002/cl2.1369. 38024780 PMC10681039

[33] M. Newson , Y. Zhao , M. E. Zein , et al., “Digital Contact Does Not Promote Wellbeing, But Face‐To‐Face Contact Does: A Cross‐National Survey During the COVID‐19 Pandemic,” New Media & Society 26, no. 1 (2024): 426–449, 10.1177/14614448211062164. 38174349 PMC10758341

[34] S. Stieger , D. Lewetz , and D. Willinger , “Face‐To‐Face More Important Than Digital Communication for Mental Health During the Pandemic,” Scientific Reports 13, no. 1 (2023): 8022, 10.1038/s41598-023-34957-4. 37198196 PMC10191089

[35] K. Nakahara and K. Yokoi , “Role of Meaningful Social Participation and Technology Use in Mitigating Loneliness and Cognitive Decline Among Older Adults,” American Journal of Occupational Therapy 78, no. 6 (2024), 10.5014/ajot.2024.050794. 39418649

[36] N. Matthews , J. B. Mattingley , and P. E. Dux , “Media‐Multitasking and Cognitive Control Across the Lifespan,” Scientific Reports 12, no. 1 (2022): 4349, 10.1038/s41598-022-07777-1. 35288584 PMC8919358

