# Supplementary material for: The Role of Social Media in Mitigating the Long‐Term Impact of Social Isolation on Mental and Cognitive Health in Older Adults During the COVID‐19 Pandemic: The HUNT Study
Source: Int J Geriatr Psychiatry. 2025 May 7;40(5):e70097. doi: 10.1002/gps.70097 (PMC12058439; doi:10.1002/gps.70097)
Supplement: Supplementary file 1 — Supporting Information S1 [file GPS-40-e70097-s001.docx]

**Supplementary tables**

**Table S1**. Multilevel mixed-effects linear regression model*, with standardized regression coefficient for mental health scores (CONOR-MHI) from baseline to follow-up.

|  | Standardized regr. coeff. (SE) | z-score | 95%CI |
| --- | --- | --- | --- |
| Change from baseline to follow-up (time) | 0.09 (0.02) | 4.59 | **0.05, 0.12** |
| Sosial isolation*¹* (time##isolation) | 0.07 (0.03) | 2.29 | **0.01, 0.13** |
| Social media *²* (time## social media)  -less than 1 hour  -1 hour or more | -0.04 (0.05)  -0.03 (0.05) | -0.94  -0.54 | -0.13, 0.05  -0.12, 0.07 |
| If isolated³ (time## social media)  -less than 1 hour  -1 hour or more | -0.04 (0.08)  -0.03 (0.09) | -0.53  -0.35 | -0.21, 0.12  -0.21, 0.15 |
| If not isolated³ (time## social media)  -less than 1 hour  -1 hour or more | -0.05 (0.05)  -0.04 (0.06) | -0.95  -0.66 | -0.16, 0.05  -0.16, 0.08 |

****The model is adjusted for* *sex, age, education level, and living situation.*

*¹ Not socially isolated as reference category.*

*²Not using social media as reference category.*

*³Analysis stratified by isolation status during the pandemic.*

**Table S2**. Multilevel mixed-effects linear regression model*, with standardized regression coefficient for cognitive function (MoCA) from baseline to follow-up.

|  | Standardized regr. coeff. (SE) | z-score | 95%CI |
| --- | --- | --- | --- |
| Change from baseline to follow-up (time) | -0.08 (0.02) | -4.97 | **-0.11, -0.05** |
| Sosial isolation*¹* (time##isolation) | -0.03 (0.03) | -0.97 | -0.08, 0.03 |
| Social media*²* (time##social media)  -less than 1 hour  -1 hour or more | 0.13 (0.04)  0.10 (0.04) | 3.33  2.31 | **0.05, 0.20**  **0.01, 0.18** |
| If isolated³ (time## social media)  -less than 1 hour  -1 hour or more | 0.15 (0.07)  0.12 (0.07) | 2.27  1.62 | **0.02, 0.28**  -0.02, 0.25 |
| If not isolated³ (time## social media)  -less than 1 hour  -1 hour or more | 0.13 (0.05)  0.09 (0.05) | 2.63  1.70 | **0.03, 0.22**  -0.01, 0.19 |

**The model is adjusted for* *sex, age, education level, and living situation.*

*¹ Not socially isolated as reference category.*

*²Not using social media as reference category.*

*³Analysis stratified by isolation status during the pandemic.*
